# Peer‐shared hobbies and quality of life in boys with autism spectrum disorder: An exploratory cross‐sectional study in Japan

**DOI:** 10.1002/pcn5.70276

**Published:** 2025-12-26

**Authors:** Masaki Seki, Hiroyuki Ogata, Tomoya Hishida, Erina Nakane, Sohei Saima, Chuichi Kondo, Toru Yoshikawa, Kasumi Miyachi, Hiroshi Ihara

**Affiliations:** ^1^ Department of Psychiatry Okute Hospital Mizunami City Gifu Japan; ^2^ Department of Advanced Internal Medicine Dokkyo Medical University Graduate School of Medicine Shimotsuga Tochigi Japan; ^3^ Department of Psychiatry Dokkyo Medical University Saitama Medical Center Koshigaya City Saitama Japan; ^4^ Department of Psychiatry Gifuminami Hospital Gifu City Gifu Japan; ^5^ Owari Public Welfare Consultation Center Nagoya City Aichi Japan

**Keywords:** adolescence, autism spectrum disorder, hobbies, peer engagement, quality of life

## Abstract

**Aim:**

In adolescence, peer relationships and leisure engagement become central to well‐being. This study aimed to characterize the hobbies of boys with autism spectrum disorder (ASD) and examine whether engagement in peer‐shared hobbies is associated with higher quality of life (QOL) across school stages (upper elementary vs. middle school).

**Methods:**

This exploratory cross‐sectional study included boys with ASD in Japan (*N* = 71). Participants reported hobby types and whether they engaged in these hobbies with friends. QOL was assessed using a validated child‐reported instrument with six subscales and a total score. Group differences (with vs. without peer‐shared hobbies) were tested for each school stage using independent‐samples *t*‐tests; effect sizes (Cohen's *d*) and both uncorrected and Holm–Bonferroni‐adjusted *p*‐values were reported. Proportions were compared with *χ* ^2^ tests.

**Results:**

Many boys expressed a desire to play with friends, but only 36.8% of upper elementary boys and 21.2% of middle school boys did so. Middle school boys with peer‐shared hobbies showed higher unadjusted total QOL than those without such hobbies (e.g., *t*(31) = 2.55, *p* = 0.016; *d* = 1.04), with small‐to‐large advantages on several subscales; however, these differences did not remain statistically significant after Holm–Bonferroni correction. Age‐group comparisons suggested increasing difficulty in achieving peer‐shared hobby engagement from late childhood to adolescence.

**Conclusion:**

Findings suggest that hobby‐based engagement with peers may be an important—but underrealized—pathway to better QOL in boys with autism. Larger, preregistered studies are warranted to confirm these associations and inform interventions that leverage shared hobbies as a scaffold for social connection and well‐being.

## INTRODUCTION

Autism spectrum disorder (ASD) is a neurodevelopmental condition characterized by two core features: persistent deficits in social communication and interaction, and restricted, repetitive patterns of behavior, interests, and activities.^1^ Prior studies reveal that the hobbies of children and adolescents with ASD are characterized by lower physical activity and a greater likelihood of solitary pursuits (e.g., playing video games alone), differing from those of typically developing children in diversity and social engagement.^2^, ^3^, ^4^ However, few studies have examined the hobby and leisure activity preferences of children with ASD in Japan.

Children and adolescents with ASD are believed to have fewer opportunities to participate in hobbies and leisure activities than typically developing children,^5^, ^6^ and although this finding derives from studies involving adults with ASD, their satisfaction with hobbies and leisure activities is considered to be lower.^7^ Among typically developing groups, participation in leisure and social activities with friends is known to buffer stress and positively impact quality of life (QOL).^8^, ^9^ However, for individuals with ASD, participation in simple social activities, such as conversing with friends, is believed to increase stress rather than buffer it.^10^ This may be due to difficulties in interpreting social cues and reduced social motivation during peer interactions, which can make such engagement more stressful than relaxing.^11^ However, prior research suggests that participation in tabletop role‐playing games (TRPGs) is associated with improvements in QOL in children with ASD.^12^ These findings imply that hobby‐based activities, rather than undifferentiated social participation, affect QOL in children with ASD. However, whether this influence stems from the hobbies themselves or from participating in hobbies with friends remains unclear.

Adolescence is a period of increased social expectations, as friendships evolve and the value of peer relationships grows with age.^13^ Adolescence is a challenging period for the mental health of typically developing children but is considered even more difficult for children with ASD.^14^ Children and adolescents with ASD wish to interact with their friends,^15^, ^16^ but they rarely meet them outside of school and are not invited to social activities,^17^ which often results in them spending much of their time alone.^18^ As friendships among children with ASD tend to weaken as they get older, and they are more likely to become isolated from their peers at school,^18^ many children and adolescents with ASD may desire social interaction but ultimately engage in activities alone.

Children with ASD are believed to have a poorer understanding of the relationship between loneliness and friendship compared to typically developing children.^15^ However, many children with ASD experience distress from loneliness and struggle with friendships as they reach adolescence.^19^ This suggests that the impact of engaging in activities with friends on their QOL may differ between late childhood (around ages 9–12) and adolescence.^13^, ^14^

To address these gaps, the present study focuses on boys with ASD in Japan and (1) characterizes the types of hobbies and leisure activities in which they engage; (2) examines whether engagement in peer‐shared hobbies is associated with higher QOL across the elementary and middle school years.

## METHODS

### Participants and eligibility

This cross‐sectional study included boys with ASD who visited the outpatient Department of Child Psychiatry at Okute Hospital between June and September 2023. Okute Hospital is located in Mizunami City in Japan. Mizunami has an estimated population of 37,000, including approximately 1700 children in the age range targeted in this study. The city has seven elementary schools and three middle schools. Most areas in this city are mountainous. Given the city's wide geographic area, some children reside far from their schools, and in some junior high schools, students commute using school bus services.

A purposive sampling strategy was employed, and 71 children were found to meet the eligibility criteria. Of these, 38 were upper elementary school students (Grades 4–6; mean age = 10.32, SD = 1.25; mean IQ = 91.81, SD = 14.13) and 33 were middle school students (Grades 7–9; mean age = 13.09, SD = 0.94; mean IQ = 93.03, SD = 12.59). All participants were diagnosed with ASD by board‐certified child psychiatrists according to DSM‐5 criteria,^1^ based on clinical interviews, developmental history, and behavioral observations. Children with comorbid intellectual developmental disorder or attention‐deficit/hyperactivity disorder were excluded. Among the 71 participants, 14 of the 38 upper elementary school boys and 8 of the 33 middle school boys were experiencing school refusal at the time of assessment. Furthermore, none of the participants in either group were receiving pharmacological treatment for depression or anxiety. No formal a priori sample size calculation was performed owing to the exploratory nature of the study; the sample comprised all eligible patients during the study period.

### Rationale for a male‐only sample

We restricted the sample to boys to reduce any gender‐related heterogeneity in leisure preferences and peer interaction patterns during late childhood and early adolescence. Furthermore, an adequate and methodologically comparable female sample could not be secured during the recruitment window. Therefore, focusing on a single gender was deemed appropriate for this exploratory study, as it improved internal validity and allowed clearer estimation of effect sizes to inform future investigations. The need to examine girls explicitly is acknowledged as an important direction for subsequent research.

### Age‐band definition and justification

Participants were a priori divided into two school‐stage groups aligned with the Japanese system: upper elementary school (Grades 4–6; approximate age 9–12 years) and middle school (Grades 7–9; approximate age 12–15 years). These stages were chosen because the transition from elementary to middle school represents a major developmental inflection in Japan. Specifically, the move to middle school is accompanied by heightened peer expectations and autonomy, increased social complexity, and structural changes in school organization and extracurricular activities. These factors plausibly alter opportunities for peer‐shared hobbies and their relation to QOL. In addition, restricting the elementary group to Grades 4–6 ensured age‐appropriate comprehension of the self‐report QOL items and comparable opportunities for hobby‐based peer engagement relative to middle school. Consistent with the study aims, this categorization allowed us to examine whether the association between shared hobbies and QOL differs between late childhood and early adolescence.

### Hobbies and leisure activities

Participants were asked to freely list up to three hobbies or leisure activities they enjoyed. For each hobby, they indicated whether it was typically performed alone or shared with friends. Sharing with friends referred specifically to engaging in the activity with school friends or peers in their everyday offline environment and was indicated by a Yes/No response.

Importantly, playing online with anonymous players or engaging solely in online multiplayer modes was not considered “sharing with friends,” as many online games allow interaction with others without involving known peers. Thus, only activities shared with identifiable friends were classified as peer‐shared hobbies in this study.

### Quality of life

QOL was assessed using the Japanese version of the KINDL‐R.^20^ For upper elementary school children, we used the Japanese Kid‐KINDL (elementary school version), and for middle school students, we used the Japanese Kid‐KINDL (junior high school version). The KINDL‐R consists of 24 items across six subscales (physical well‐being, emotional well‐being, self‐esteem, family, friends, and school functioning), with responses rated on a 5‐point Likert scale. Subscale scores were transformed into a 0–100 scale, with higher scores indicating better QOL. The reliability and validity of the Japanese versions have been previously confirmed.^21^, ^22^

### Ethics approval and informed consent

The study protocol was approved by the Okute Hospital Ethics Committee (Approval No. R5‐1). Written informed consent was obtained from all parents or legal guardians, and assent was obtained from all participating children.

### Missing data

Among the 71 participants, KINDL‐R data were available for 38 elementary school students and 33 middle school students. In the middle school group, two participants (2.8% of the total sample) had partial missing responses on the Friends and School subscales. Therefore, the valid number of cases varied slightly across subscales (*N* = 33 for Physical, Emotional, Self‐esteem, Family, and Total; *N* = 32 for Friends and School). After listwise deletion, 31 complete cases remained for multivariable comparisons.

### Statistical analysis

Descriptive statistics (means, standard deviations, ranges, and frequencies) were calculated for demographic variables, hobby‐related variables, and each KINDL‐R subscale. Group differences in QOL scores between children who engaged in hobbies with friends and those who did not were examined separately for elementary and middle school students. Independent‐samples *t*‐tests with Welch's correction were used to account for potential variance heterogeneity. In addition to *p*‐values, effect sizes (Cohen's *d*) with 95% confidence intervals were reported to aid interpretation.

To address the risk of Type I error due to multiple testing across six subscales and the total score, both uncorrected and Holm–Bonferroni‐adjusted *p*‐values were presented. This dual reporting approach followed the recommendations for exploratory studies^23^, ^24^; it allows readers to evaluate both unadjusted findings and results that remain robust after correction.

For categorical variables (e.g., proportions of children wishing to engage in hobbies with friends), chi‐square or Fisher's exact tests were conducted as appropriate. To assess potential outliers and illustrate score distributions, supplementary visualizations (box‐and‐whisker plots) were generated.

All analyses were conducted using IBM spss Statistics for Mac, version 29.0.2.0. Statistical significance was set at *p* < 0.05 (two‐tailed). Given the limited sample size, all results were considered preliminary. Effect sizes were reported to indicate the magnitude of observed differences; however, replication with larger samples is required in the future.

## RESULTS

### Hobbies of boys with ASD

The most common hobbies among upper elementary school boys with ASD were playing video games (89.47%), physical activities such as sports and outdoor play (42.11%), watching videos on YouTube (28.95%), drawing illustrations and comics (18.42%), reading comics (10.52%), and reading books (10.52%).

**Figure 1:**
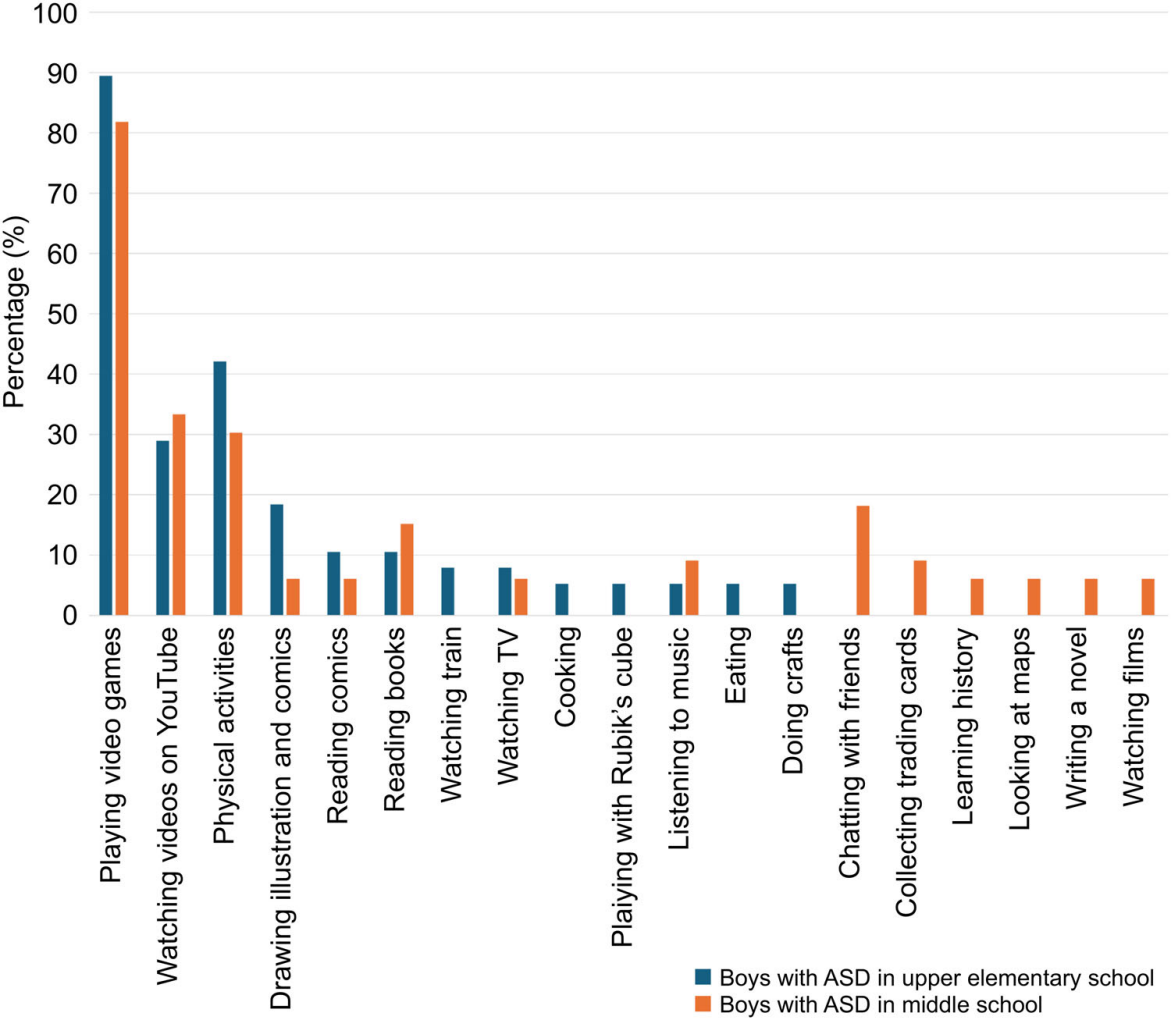
Comparison of hobby categories between upper elementary and middle school boys with autism spectrum disorder (ASD). This figure presents the percentage of students in each school group who reported engaging in each hobby category. Among upper elementary school boys, the most common hobbies were playing video games (89.5%), engaging in physical activities such as sports and outdoor play (42.1%), and watching videos on YouTube (28.9%). Among middle school boys, the most common hobby was playing video games (81.8%), followed by watching videos on YouTube (33.3%), and physical activities (30.3%). Some also listed chatting with friends (18.2%) as a hobby.

The most common hobbies among middle school boys with ASD were playing video games (81.81%), watching videos on YouTube (33.33%), physical activities such as sports and outdoor play (30.3%), chatting with friends (18.18%), and reading books (15.15%). “Chatting with friends” was not reported as a hobby among upper elementary school boys and was observed only in middle school boys (Figure 1).

Because playing video games was the most frequently mentioned hobby in both groups, we briefly analyzed the games reported. In the elementary school group, Minecraft (55.26%), Splatoon (23.68%), and Pokémon (23.68%) were most commonly listed. Minecraft is a sandbox building game, Splatoon is a team‐based shooter game, and Pokémon is a role‐playing game.

In the middle school group, Minecraft (21.21%), Splatoon (15.15%), and Fortnite (15.15%) were listed most frequently. Fortnite is a battle‐royale shooter game that can be played online. The middle school group also reported a wider variety of individual games, with 46 different titles mentioned, compared to 31 titles reported by the upper elementary school group. These titles included games that allow online competitive or cooperative play with friends.

### Boys with ASD who wished to play with friends

A chi‐square test was conducted to examine group differences in the proportion of boys with ASD who expressed a desire to play with friends. In the elementary school group, 78.9% reported wanting to play with friends, compared with 66.6% in the middle school group. This difference was not statistically significant, *χ* ^2^(1, *N* = 71) = 0.80, *p* = 0.37.

### Boys with ASD who wished to play with friends and were able to do so

A chi‐square test was conducted to examine group differences in the proportion of boys with ASD who reported actually playing with friends. Although a higher percentage of upper elementary school boys (36.8%) than middle school boys (21.2%) reported engaging in hobbies with friends, this difference was not statistically significant, *χ* ^2^(1, *N* = 71) = 1.39, *p* = 0.24.

### Quality of life scores

Independent‐samples *t*‐tests were conducted to compare the KINDL‐R subscales and total scores between boys who engaged in hobbies with friends and those who did not. Among upper elementary school boys with ASD, there were no significant differences in any QOL dimension between those who engaged in hobbies with friends and those who did not (Table 1).

**Table 1:** Comparison of quality of life between boys with autism spectrum disorder (ASD) who played with and without friends in upper elementary school.

Subscale	Playing with friends (*n* = 14)	Not playing with friends (*n* = 24)	*t*(df)	Uncorrected *p*	Holm–Bonferroni corrected *p*	Cohen's *d*	95% CI of *d*
	*M* (SD)	*M* (SD)					
Physical	75.14 (19.74)	76.00 (14.70)	−0.15 (36)	0.879	1.000	−0.05	[−0.71, 0.61]
Emotional	83.57 (16.83)	79.38 (18.00)	0.71 (36)	0.483	0.966	0.24	[−0.42, 0.90]
Self‐esteem	59.07 (29.66)	62.09 (23.82)	−0.34 (35)	0.736	1.000	−0.12	[−0.78, 0.55]
Family	79.57 (18.21)	80.58 (14.17)	−0.19 (36)	0.850	1.000	−0.06	[−0.72, 0.59]
Friends	74.36 (18.88)	65.91 (21.28)	1.22 (35)	0.231	0.966	0.41	[−0.26, 1.08]
School	58.21 (22.50)	63.39 (18.35)	−0.76 (35)	0.450	0.966	−0.26	[−0.92, 0.41]
Total QOL	71.57 (13.85)	69.71 (13.84)	0.40 (36)	0.691	1.000	0.14	[−0.53, 0.79]

*Note*: Effect sizes are reported as Cohen's *d* with 95% confidence intervals. Holm–Bonferroni correction was applied across all comparisons; no differences remained statistically significant after correction.

Abbreviations: M, mean; QOL, quality of life; SD, standard deviation.

Among middle school boys with ASD, before correcting for multiple comparisons, significant group differences were observed in the self‐esteem (*t*(31) = 2.77, *p* = 0.009) and friends subscales (*t*(30) = 2.29, *p* = 0.029) and total QOL (*t*(31) = 2.55, *p* = 0.016) between boys who shared hobbies with friends and those who did not. After applying the Holm–Bonferroni adjustment across the six subscales and total score (seven tests), none of these differences remained statistically significant. Large effect sizes were obtained for self‐esteem (*d* = 1.13), friends (*d* = 0.94), and total QOL (*d* = 1.04); moderate effect sizes for physical health (*d* = 0.70) and emotional well‐being (*d* = 0.62); and small‐to‐moderate effect sizes for family (*d* = 0.38) and school (*d* = 0.48) (Table 2).

**Table 2:** Comparison of quality of life between boys with autism spectrum disorder (ASD) who played with and without friends in middle school.

Subscale	Playing with friends (*n* = 8)	Not playing with friends (*n* = 25)	*t*(df)	Uncorrected *p*	Holm–Bonferroni corrected *p*	Cohen's *d*	95% CI of *d*
	*M* (SD)	*M* (SD)					
Physical	78.25 (18.81)	62.40 (23.83)	1.71 (31)	0.097	0.388	0.70	[−0.12, 1.51]
Emotional	86.00 (14.02)	74.20 (20.37)	1.52 (31)	0.139	0.556	0.62	[−0.20, 1.42]
Self‐esteem	74.38 (26.29)	41.60 (29.89)	2.77 (31)	0.009*	0.063	1.13	[0.27, 1.96]
Family	76.50 (23.90)	67.28 (24.27)	0.94 (31)	0.355	1.000	0.38	[−0.42, 1.18]
Friends	81.38 (18.85)	54.50 (31.10)	2.29 (30)	0.029*	0.203	0.94	[0.10, 1.76]
School	58.63 (26.00)	47.79 (21.50)	1.17 (30)	0.250	1.000	0.48	[−0.33, 1.28]
Total QOL	75.75 (17.95)	57.04 (18.08)	2.55 (31)	0.016*	0.112	1.04	[0.19, 1.87]

*Note*: Effect sizes are reported as Cohen's *d* with 95% confidence intervals. Holm–Bonferroni correction was applied across the six subscales and the total score. After correction, no group differences remained statistically significant.

Abbreviations: M, mean; QOL, quality of life; SD, standard deviation.

* *p* < 0.05 (uncorrected).

**Figure 2:**
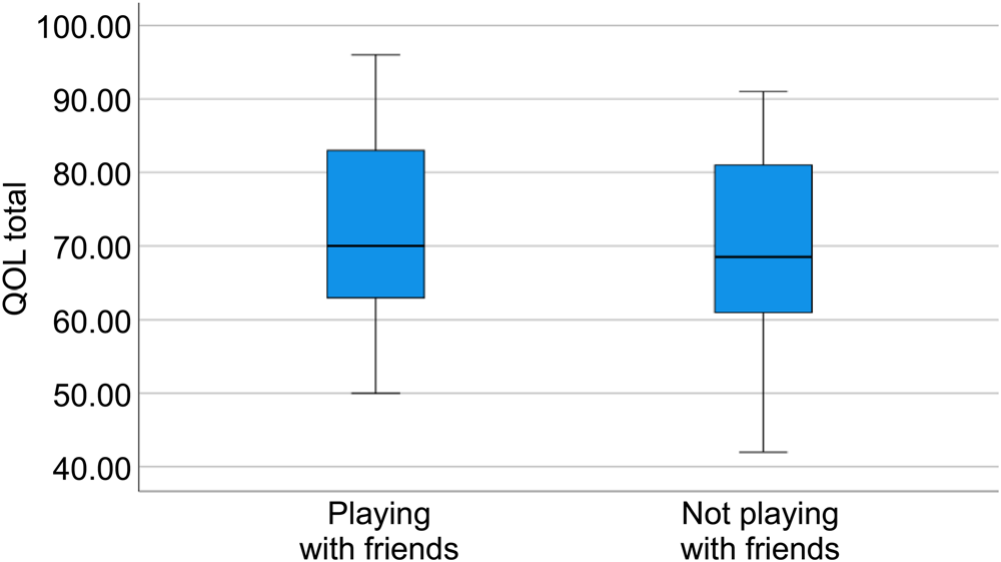
Box‐and‐whisker plot of total quality of life (QOL) scores comparing boys who engaged in hobbies with friends and those who did not, in the upper elementary group. The horizontal line denotes the median; the box spans the interquartile range (IQR); the whiskers extend to the most extreme values within 1.5 × IQR of the quartiles. Outliers were identified using the 1.5 × IQR rule and retained in the analysis. “Engaging in hobbies with friends” denotes co‐activity (doing/playing together), not merely sharing the same interests.

**Figure 3:**
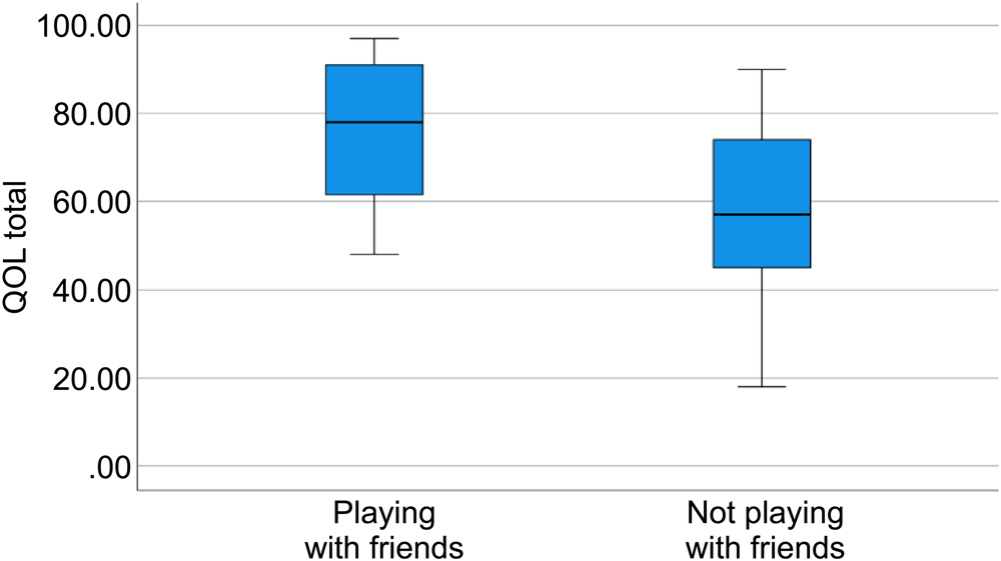
Box‐and‐whisker plot of total quality of life (QOL) scores comparing boys who engaged in hobbies with friends and those who did not, in the middle school group. The horizontal line denotes the median; the box spans the interquartile range (IQR); the whiskers extend to the most extreme values within 1.5 × IQR of the quartiles. Outliers were identified using the 1.5 × IQR rule and retained in the analysis. “Engaging in hobbies with friends” denotes co‐activity (doing/playing together), not merely sharing the same interests.

Figures 2 and 3 present the box‐and‐whisker plots of the comparison of total QOL scores between boys who shared hobbies with friends and those who did not, stratified by school level.

## DISCUSSION

### Hobbies preferred by elementary and middle school boys with ASD

Playing video games was the most preferred hobby among boys with ASD, cited by 89.5% of upper elementary school boys and 81.8% of middle school boys. Although a direct comparison is not possible owing to differences in survey methods, this percentage is higher than that reported by Russell et al. (23.3%).^2^ One possible reason for this is the widespread popularity of home video game consoles in Japan. For instance, the Nintendo Switch series has sold over 33 million units in Japan.^25^ It is likely that many Japanese children own a Nintendo Switch as their personal game console. Furthermore, many contemporary games are played on smartphones; smartphone ownership rates among upper elementary and middle school students in Japan are 42% and 79%, respectively, suggesting the widespread smartphone adoption as a contributing factor.^26^

In a previous study, Minecraft and racing games were the most frequently preferred video games among boys with ASD, while racing games were not mentioned in the responses of typically developing children.^27^ In this survey, Japanese upper elementary school boys with ASD listed Minecraft, Splatoon, and Pokémon, and middle school boys listed Minecraft, Splatoon, and Fortnite. This discrepancy may reflect cultural or contextual factors that shape gaming preferences, including regional trends, available gaming platforms, and peer culture. As game popularity changes across countries and over time, such contextual influences may explain differences between previous findings and those observed in the present study. The game titles identified in this study are popular among children of the same age in Japan, and no specific genres unique to upper elementary or middle school boys with ASD were observed.

Furthermore, the titles listed above are games that allow players to cooperate or compete online with their friends. This suggests that, even in geographically dispersed communities such as those in Mizunami City, online gaming can function as a means of engaging in shared activities with peers.

### Relationship between playing with friends and QOL

A notable finding is the gap between children's desire to play with friends and their actual engagement. While 78.9% of upper elementary boys and 66.6% of middle school boys reported wanting to play with friends, only 36.8% and 21.2%, respectively, reported doing so. This discrepancy reflects a pattern observed in prior research indicating that children and adolescents with ASD often wish to form friendships but nonetheless spend considerable time alone.^15^, ^17^

Another important finding was that, in the middle school group, boys who shared hobbies with friends showed higher unadjusted scores for self‐esteem, friends, and total QOL than those who did not. Although these differences did not remain statistically significant after correction, the large effect sizes suggest that peer‐shared hobby engagement has a meaningful association with QOL.

As this was a cross‐sectional study, causal relationships could not be inferred. Several factors may explain why sharing hobbies with friends did not result in QOL differences in the upper elementary school group. Children with ASD have been reported to show weaker links between loneliness and friendship and to place less emphasis on intimacy or affection in friendships.^15^ They also tend to exhibit lower social motivation and are less influenced by others' evaluations.^11^ These characteristics may reduce the negative emotional impact of solitary leisure activities; thus, not engaging in hobbies with friends may not necessarily lead to lower QOL in upper elementary school boys.

In contrast, friendship‐related challenges tend to increase during adolescence. Children with ASD often experience heightened awareness of social isolation, conflict, and victimization as they grow older.^19^, ^28^ In adolescents with ASD, friendship quality is known to be associated with mental health outcomes such as depression and loneliness.^6^, ^29^ Moreover, friendships in this population often develop around shared interests,^28^, ^30^ making hobby‐based peer engagement a potentially important pathway to enhance well‐being. This may help explain why middle school boys who engaged in hobbies with friends tended to show higher QOL.

Notably, no statistically significant differences were detected after multiple comparison correction; several factors may have attenuated these group differences. The relatively small sample size likely reduced statistical power. Moreover, QOL is a multidimensional construct influenced by family functioning, school climate, physical health, and social support, and variability in these domains may have masked the specific contribution of shared hobbies. Furthermore, engagement in shared hobbies was assessed using a single dichotomous item (Yes/No), which did not capture the frequency or quality of peer interactions, potentially reducing sensitivity to true differences.

### Clinical and educational implications

This study contributes novel insights into how engagement in shared hobbies with friends impacts QOL in boys with ASD, a population that is underrepresented in research. The primary finding was that although boys with ASD in both elementary and middle school expressed a desire to engage in hobbies with friends, only a small proportion actually did so. Furthermore, the results revealed that as children with ASD enter adolescence, engagement in leisure activities involving shared hobbies with peers may play an important role in well‐being.

These findings have important clinical and policy implications. From a clinical perspective, structured interventions that facilitate peer engagement through hobbies—such as social skills groups based on shared interests or community‐based leisure programs—may enhance QOL in adolescents with ASD. Schools and local governments should consider creating inclusive environments and after‐school programs where students with ASD can naturally interact with peers through enjoyable and familiar activities, such as gaming or outdoor play.

In Japan, practice‐based implementations using TRPGs and conversations centered on hobbies have been reported^12^, ^31^; however, empirical evidence in this area remains scarce. Future research should examine the longitudinal effects of shared hobby activities on social development and mental health in ASD populations.

### Limitations

Several limitations must be noted in the context of this study. First, the sample size was modest, which limited statistical power and increased susceptibility to Type I and Type II errors. Although effect sizes were substantial, larger samples are needed to confirm these associations. Second, the study focused exclusively on boys; given prior evidence of gender differences in socialization and leisure patterns, future research should include girls with ASD. Third, the cross‐sectional design precludes conclusions about developmental trajectories; longitudinal studies are necessary to clarify how hobby engagement and its relation to QOL evolve throughout adolescence. Fourth, the present analyses did not differentiate between types of hobbies (e.g., sports, creative activities, and video games) in relation to QOL outcomes. Although exploratory comparisons suggested that shared engagement itself may be beneficial, prior evidence indicates that certain activities—such as physical play—may be more strongly associated with peer interaction than others.^2^, ^3^ Future research with larger samples should examine how specific hobby categories contribute distinctly to QOL in youth with autism. Finally, the reliance on self‐ and parent‐reports may have introduced reporting bias, underscoring the need for multi‐informant and observational methods.

### Conclusions

This study indicates that shared hobbies may be associated with higher QOL in boys with ASD in Japan, particularly in domains related to peer relationships and self‐worth. While statistical significance did not withstand correction, effect sizes pointed to potentially meaningful benefits that merit further investigation. Future research should build on these preliminary findings by examining larger and more diverse samples, considering gender differences and developmental stages. Such research can help design interventions that harness shared hobbies as a pathway to improved social participation and well‐being for children with ASD.

## AUTHOR CONTRIBUTIONS

**Masaki Seki**: Conceptualization; methodology; data curation; writing—original draft. **Hiroyuki Ogata**: Writing—review and editing. **Tomoya Hishida**: Data curation; writing—review and editing. **Erina Nakane**: Writing—review and editing. **Sohei Saima**: Writing—review and editing. **Chuichi Kondo**: Writing—review and editing. **Toru Yoshikawa**: Writing—review and editing. **Kasumi Miyachi**: Writing—review and editing. **Hiroshi Ihara**: Supervision; writing—review and editing.

## CONFLICT OF INTEREST STATEMENT

The authors declare no conflicts of interest.

## ETHICS APPROVAL STATEMENT

The study protocol was approved by the Okute Hospital Ethics Committee (Approval No. R5‐1).

## PATIENT CONSENT STATEMENT

Written informed consent was obtained from all parents or legal guardians, and assent was obtained from all participating children.

## CLINICAL TRIAL REGISTRATION

Not applicable.

## DECLARATION OF GENERATIVE AI IN SCIENTIFIC WRITING

The authors used a generative AI tool (ChatGPT, OpenAI) solely for English language editing.

## Data Availability

The datasets generated and analyzed during the current study are available from the corresponding author on reasonable request.
